# Sense of coherence and health in women: a 25-year follow-up study

**DOI:** 10.1186/s12905-023-02834-x

**Published:** 2023-12-13

**Authors:** Marie Bladh, Gunilla Sydsjö, Lisa Ekselius, Eva Vingård, Sara Agnafors

**Affiliations:** 1 Department of Biomedical & Clinical Sciences (BKV), BKH/Obstetrics & Gynaecology, Faculty of Medicine and Health Sciences, Linköping University, Linköping, SE-58185 Sweden; 2 Women’s Mental Health during the Reproductive Lifespan, Department of Women’s and Children’s Health, Uppsala University, Uppsala, SE-75185 Sweden; 3 Department of Occupational and Environmental Medicine, Uppsala University, Uppsala, SE-75185 Sweden; 4 Department of Research, Södra Älvsborgs Hospital, Borås, Sweden

**Keywords:** Sense of coherence, Health, Women, Longitudinal

## Abstract

**Background:**

Health and Sense of Coherence (SOC) has been shown to be intertwined and argued to have a reciprocal relationship. The theory of SOC implies relatively stable scores during adulthood, however there are few longitudinal studies on the association between SOC and mental and somatic health. The main aim of the present study was to examine how SOC and self-rated health (SRH) are related during 25 years of follow-up.

**Methods:**

Using paper questionnaires distributed by postal services, 415 mothers were followed from childbirth and 25 years prospectively. SOC was measured at three, 12 and 25 years after inclusion. Self-reports on health status were obtained at the 25-year follow-up. The association between SOC and self-reported health as well as the effect of sociodemographic factors and experience of stressful life events was assessed through regression models.

**Results:**

SOC scores increased between three and 12 years after inclusion, and slightly decreased at the 25-year follow-up. Women of good health had a higher SOC-score at all three measurements compared to women of poor health. Multiple logistic regression showed that the likelihood of reporting good health increased with the number of times the women had reported SOC-scores above the 75th percentile. Moreover, women who had not been through a divorce were close to 60% more likely to report good health compared to women who had been through a divorce, whereas women not reporting stressful life events during the past two years were more than twice as likely to report good health. Symptoms below cut-off for postpartum depression and not having been through a divorce were associated with SOC scores above the 75th percentile.

**Conclusion:**

This 25-year follow-up study of a cohort of women reports good stability of SOC assessments in the vast majority of women. There was a stronger and more stable SOC in women with better health. The findings are in line with other studies on the predictive value of SOC and self-perceived health.

## Introduction

Health is a complex entity both from a biomedical view based on biology, pathology, and physiology and from a humanistic view based humanistic and social concepts. Nordenfeldt has presented a definition of health. “Health is a person’s ability, given standard circumstances, to achieve his or hers vital goals and thus realize minimal happiness” [1]. Additionaly, Medin and Alexandersson (2000) identified three main concepts of health in a literature review; (1) Health as the absence of illness, (2) Health as a resource, a strength, and (3) Health as the state of being in balance [2].

In 1979 Aaron Antonovsky introduced the salutogenic model with the question why some people stay well despite stressful situations and hardship. Within the salutogenic perspective Antonovsky developed the theory about sense of coherence (SOC). The SOC model includes the three components comprehensibility, manageability, and meaningfulness. A high SOC has been used as a marker for good mental and physical health [3] and for resources to be used to move in a health-promoting direction. A high SOC has been found to promote better lifestyle choices promoting a better self-perceived health [4] and better coping skills when exposed to stressful events [5, 6]. Thus, stressful life events and life situations where personal control strategies are inadequate or dependent on other people have been found to be associated with a lower SOC [6]. There are also reports on reciprocal relationship, that good health itself is an indicator for experiencing a high sense of coherence [7]. Antonovsky argues that SOC develops through early adulthood, but then stabilizes and alters only in response to stressful life events [5]. However, the stability of SOC over time has been questioned, and found to vary with experience of life events, where women with a high score have been found to be more likely to exhibit a stable score when followed during a longer period [8, 9].

Studies have shown that adolescents with a low SOC were more likely to express depression and mental ill-health, as well as lower levels of self-rated health [10]. Also, in a recent study among women who had just given birth, it was found that women with a higher SOC were less likely to experience depressive symptoms after childbirth compared to women who scored lower on SOC [11]. Moreover, a strong SOC have been found to be associated with reduced risk for anxiety in women living in deprived conditions, where an index for deprivation was derived based on e.g., overcrowded living space and rate of unemployment [12]. SOC has also been found to impact the intention of early retirement in both men and women [13]. Furthermore, several studies on care professionals have indicated that work-family conflicts/stress negatively affects health, with an increased risk for mental ill health and that a stronger SOC may reduce the negative impact of work-family conflicts/stress [14].

Longitudinal studies measuring self-perceived mental health as well as somatic health and its association to SOC are few. However, a recent longitudinal study, with follow-ups of both men and women 10 and 20 years after inclusion, found an inversed relationship between SOC and self-perceived health as well as health as assessed by physicians [15]. In a study investigating the association between SOC and health over a 4 year period, SOC was found to predict sickness leave in women, but not in men [16]. Also, some evidence has been presented showing that not having been through a divorce and educational level are associated with a higher level of SOC, whereas the studies have found both a negative as well as positive association between age and SOC [17, 18].

Thus, the aim of the current study the association(s) between sociodemographic background factors, life events, SOC, self-rated health as well as the symptom stability of SOC across several measurements.

More specifically, in this study it was hypothesized that women with high SOC report a better health compared to women with a lower SOC, and that a high SOC at several measurements is associated with a better self-reported health. Also, it was hypothesized that self-rated health was inversely associated with symptoms of depression at time of childbirth as well as exposure to stressors such as having immigrated to Sweden traumatic life events, work conflicts and divorce since childbirth.

It was also hypothesized that increasing age and a higher level of education were positively associated with SOC.

## Methods

### Study cohort

**Fig. 1:**
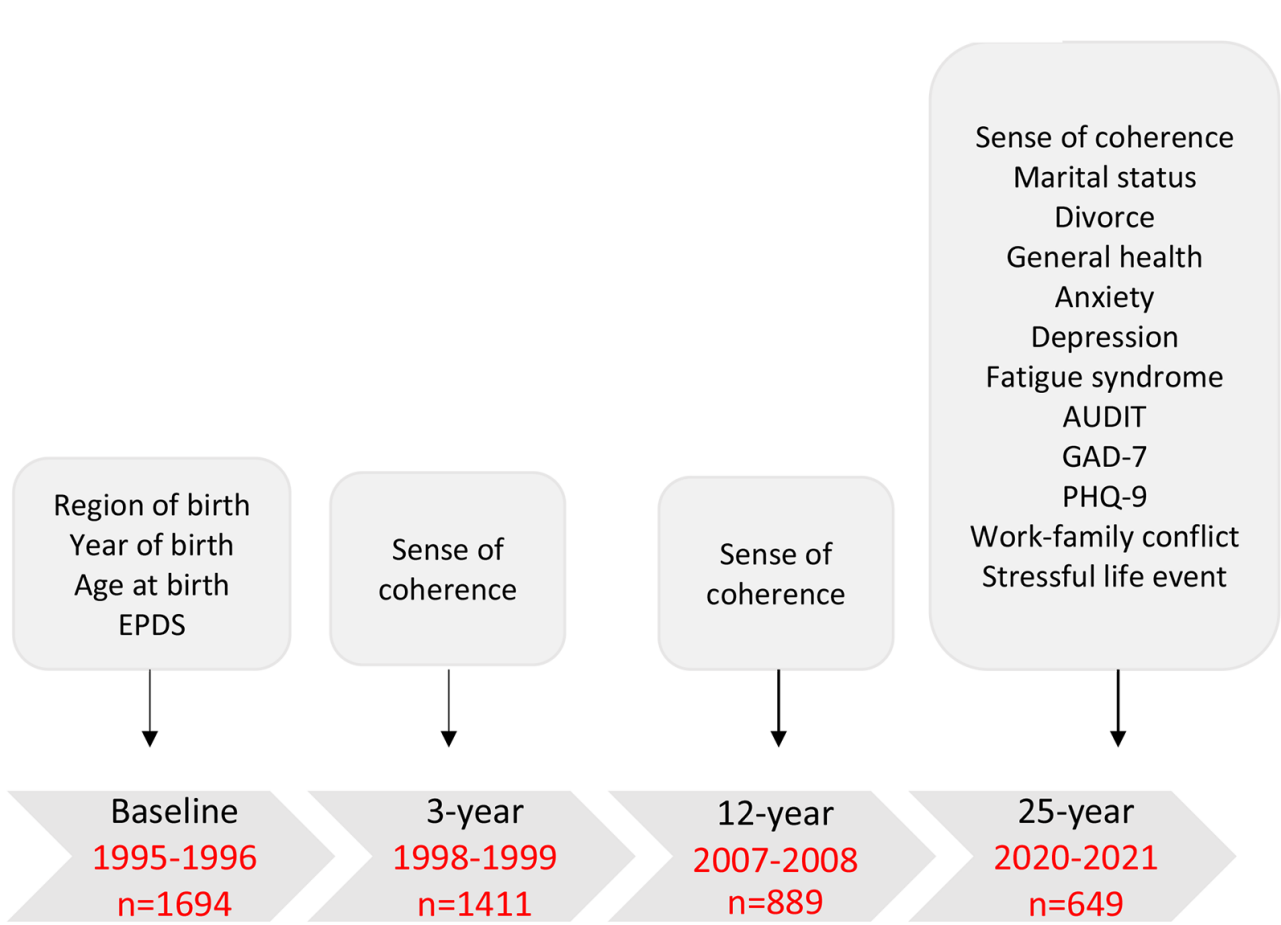
Description of measurements at the four time points included in the study

*The South East Sweden Birth Cohort study (SESBiC)* study was initiated in 1995 with the purpose of early identification of children (and women) at risk for dysfunctional development. All mothers who gave birth during a period of 20 consecutive months during 1995–1996 in five adjacent municipalities in the South East of Sweden were invited to take part in the study. Mothers (n = 1694) of 1723 children (88% of invited) accepted and were enrolled in the study. Information has been collected at totally four time points, at inclusion (baseline), and three, 12 and 25 years after inclusion. At all four time points, a study specific questionnaire has been used to collect data where some of the psychometric instruments have been used in several follow-ups to ensure comparisons over time, In Fig. 1 information from each time point used in the current study is displayed, including years when each follow-up was performed.

One of the risk groups previously identified in the SESBiC study was women with symptoms of postpartum depression (PPD). In the SESBiC study, symptoms of PPD increased the risk for subsequent depressive symptoms 12 years later and for behavioral and emotional problems in offspring at age three (Agnafors et al. 2013; Agnafors et al. 2016).

### Study population

All women who participated in the baseline study were sent an envelope with an information letter regarding the 25-year follow-up and contact information to investigators if additional information was desired. Included in the envelope were a consent form as well as the questionnaire, and a pre-stamped and pre-addressed envelope to return the signed consent form and the questionnaire. All participants had to sign and return an informed consent to being included in the 25-year follow-up. Upon completion of the questionnaire the participants received a lottery ticket (cost €3).

Of the 1694 mothers included at baseline, a total of 649 mothers participated in the follow-up approximately 25 years after they gave birth to their index child, resulting in a participation rate of 38% (649/1694 = 38%). Participation rates at the two previous follow-ups were 83% (1411/1694 = 83%) at the 3-year follow up and 52% (889/1694 = 52%) at the 12-year follow-up. Among the 649 mothers participating at the 25-year follow-up, 586 and 601 mothers had completed the SOC questionnaire in the previous follow-ups at three years and 12 years after childbirth, respectively, of which 415 mothers had participated in both previous follow-ups. Thus, the final study population consisted of 415 women which corresponds to an inclusion rate of 64% (415/649 = 64%) of those who participated in the 25-year follow-up. However, it only represents 415/1694 = 24.5% of the baseline population.

### Drop-out analysis

A comparison of baseline characteristics between the 415 women included in the study and the remaining 1279 who were not part of the study has been performed. These analyses revealed that non-participants to a greater extent were immigrants (13.2% vs. 5.1%), more often scored above cut-off of 10 on EPDS (13.5% vs. 7.3%), and had a greater exposure to total life stress (10.1% vs. 3.1%) at baseline (all p-values < 0.001).

### Data collection

#### Instruments and questionnaire

In this longitudinal study, a study specific questionnaire was used 25 years after inclusion, to collect information on the women’s employment, level of education, and civil status. Information was also collected regarding the women’s perceived health, presence of somatic and mental health problems, and stressful life events during the past two years. In addition, information from previous follow-ups (baseline, 3-year follow-up, and 12-year follow-up) were included in the study.

#### The edinburgh postnatal depression scale (EPDS)

In order to investigate women’s self-perceived symptoms of depression the Edinburgh Postnatal Depression Scale [19] was used at baseline. EPDS is a self-report form comprising ten items scored 0–3 measuring symptoms of depression during the preceding week. The form is designed to screen for postpartum depression in community samples and has been widely used. In accordance with previous studies with screening purpose, a cut-off level of 12 was used [20].

#### Sense of coherence (SOC)

The Sense of coherence scale, developed by Antonovsky [5, 21, 22], is focused on one’s ability to identify resources for health and well-being. SOC is the capability to manage whatever the situation demands in life to perceive life as comprehensible, manageable, and meaningful. The SOC scale has a long and a short version. The shorter version of SOC, comprising 13 questions, was used in the follow-ups at three and 12 years after childbirth whereas the full version of 29 questions was used in the last follow-up. To achieve comparable scales, the 13 questions from the shorter version were extracted from the full SOC version and total scores were computed in the same way as in the two previous follow-ups. The short version consists of 13 items, with total scores ranging from 13 to 91 points. The respondents rated items on a 7-point scale (1 = never; 7 = very often), where higher scores represent greater SOC. In the current study Cronbach’s alpha was 0.824 at the 12-year follow-up, and 0.876 at the 25-year follow-up. SOC at each of the three measurements (three, 12 and 25 years) were coded into < = 75th percentile and > 75th percentile. Moreover, a variable using the information on all three measurements was defined as Never > 75th percentile, Mixed above or below the 75th percentile, and Always > 75th percentile.

#### Self-rated health

At the 25-year follow-up, self-rated health was assessed by one general question from SF-36 [23]. “In general, how would you define your health?” with five possible answers *excellent, very good, good, less good*, and *poor*.

#### The patient health questionnaire (PHQ-9) 25 years

*PHQ-9* is an instrument constructed of nine questions where the respondents were asked to assess their depressive symptoms [24] at the 25-year follow-up. Each question is answered on a 4-point Likert scale, ranging from 0 = not at all to 3 = almost every day. Thus, the maximum total score is 27 where 0–4 points s considered *no to minimal depression*, 5–9 points signifies *mild depression*, 10–14 is *moderate depression*, 15–19 *moderate to severe depression*, and 20–27 as *severe depression*. Cronbach’s alpha was 0.914.

#### Generalized anxiety disorder questionnaire (GAD-7)

*GAD-7* was used in the 25-year follow-up to screen for and assess the severity of generalized anxiety disorder [25–27]. It comprises seven questions, each question scored between zero and three (0 = not at all, 1 = several days, 2 = more than half the days, and 3 = nearly every day), resulting in a possible maximum score of 21. When screening for GAD a cut-off of ≥ 10 is recommended (Mild = 5, moderate = 10, severe = 15). In accordance, GAD was divided into total scores of 0–9 and 10 or more. Cronbach’s alpha was 0.898.

#### Stressful life event

The questionnaire at the 25-year follow-up contained a question where the women were asked to state the presence of four life events (illness or accident of close husband, wife, partner or child, death of close husband, wife, partner or child, death of close relatives or close friend(s), worsened household finances) during the past two years and the effect it had on herself. The presence of an event was scored as one otherwise zero, resulting in a total score ranging between 0 and 4.

#### Alcohol use disorders identification test (AUDIT)

AUDIT, developed by the World Health Organisation (WHO) [28], is a comprehensive alcohol harm screening tool., and was filled out by the women at the 25-year follow-up. AUDIT comprises ten items asking direct questions regarding alcohol covering three different areas. Questions 1–3 covers the consumption habits, questions 4–6 covers possible dependence on alcohol while questions 7–10 ask about harmful alcohol use. Each question is scored between 0 and 4, yielding a maximum total score of 40. According to Swedish standard a score of ≥ 6 is used for identifying hazardous or harmful alcohol consumption, ≥ 14 as an indicator for possible alcohol dependence, whereas ≥ 18 is used to identify likely severe dependence and harm [29, 30]. The AUDIT-score was divided into a total score of 0–5 or 6 or more.

### Definition of good health

*Good health* 25 years after inclusion was defined as self-reported health said to be excellent or very good while also having a total score ≤ 4 on the PHQ-scale whereas *Poor health* was defined as stating a health to be less good or poor, or having a totals score ≥ 5 on the PHQ-scale. According to this definition, 182 (45.6%) women were classified as having good health, and 217 (54.4%) as having poor health. A total of 16 women had not provided their self-reported health and are thus coded as missing.

#### Sociodemographic background factors

*Region of birth* was classified as either born in Sweden or outside of Sweden. *Marital status* was divided into single, married/cohabiting or divorced/widowed. An indicator variable for ever having been through a *divorce* during the study period was created and defined as yes or no. Presently being diagnosed with or being treated for *anxiety, depression or fatigue syndrome* were all, as separate variables, defined as yes/no. Indicator variables for work-family conflict at 0–5 years, 6–12 years, and 13–20 years after inclusion were all dichotomized into yes/no.

#### Statistics

Descriptive information on continuous data is presented as median, minimum, and maximum values, whereas categorical data are presented by numbers (n) and percent (%).

Bivariate analyses included cross tabulations of health status and socio-demographic and medical factors. These analyses were evaluated using Pearson’s chi-square where the expected cell count was five or more. In case the expected cell count was below five, Fisher’s exact test was used. Single and multiple logistic regression models on health outcome was performed to evaluate independent factors’ potential effects on the outcome. Repeated measures ANOVA was used to evaluate the development of scores on SOC and its association to socio-demographic and medical history. All analyses were performed using IBM SPP, version 28 (IBM Inc., Armonk, NY, USA). Statistical significance was defined as p-value < 0.05 (two-sided).

## Results

### Descriptive analyses

The women included in the study were on average 53–54 years of age and the majority were born in Sweden (95%), no differences in age or region of birth were found with respect to health status nor with SOC-score. However, women reporting good health more often had a college/university degree compared to women reporting poor health (59.9% vs. 44.7%), Table 1. Moreover, women who reported good health and women with a SOC score above the 75th percentile had less often been through a divorce (32.4% vs. 46.1% and 29.4% vs. 44.4%, respectively) though the estimated effect size was rather small (both < 0.3), and exhibited symptoms of anxiety and/or depression to a lesser extent (3.3% vs. 18.9% and 3.9% vs. 15.0%, respectively) compared with women of poor health or women with SOC scores < = 75th percentile. As for having been through a divorce, the effect sizes for having exhibited symptoms of anxiety and/or depression was rather small. Self-reported good health and SOC above the 75th percentile at 25-year follow-up were all more prevalent among women who had not experienced collision between family and work demands during the child’s upbringing, Table 1. Moreover, not reporting experience of stressful life events during the past two years, was found to be less common among women reporting good health.

**Table 1:** Socio-demographic and medical information reported by health status and sense of coherence (SOC) 25 years after inclusion

	Health status*			SOC 25 years		
	Poor	Good		<=75th percentile	> 75th percentile	
	n (%)	n (%)	p-value (Effect size)	n (%)	n (%)	p-value (Effect size)
Age at follow-up, mean/SD	53.66/4.76	53.63/3.98	0.943	53.51/4.45	53.94/4.45	0.401
Region of birth			0.668			0.809
Sweden	206 (94.9)	171 (94.0)	(0.021)	294 (93.9)	97 (95.1)	(0.022
Outside Sweden	11 (5.1)	11 (6.0)		19 (6.1)	5 (4.9)	
Marital status			0.464			0.319
Single	22 (10.1)	12 (6.6)	(0.064)	27 (8.6)	8 (7.8)	(0.075)
Married/cohabiting	177 (81.6)	155 (85.2)		256 (81.8)	89 (87.3)	
Divorced/Widow	18 (8.3)	15 (8.2)		30 (9.6)	5 (4.9)	
College/university			0.003			0.793
No	120 (55.3)	73 (40.1)	(0.151)	154 (49.2)	48 (47.1)	(0.018)
Yes	97 (44.7)	109 (59.9)		159 (50.8)	54 (52.9)	
Any divorce			0.006			0.008
No	117 (53.9)	123 (67.6)	(0.139)	174 (55.6)	72 (70.6)	(0.131)
Yes	100 (46.1)	59 (32.4)		139 (44.4)	30 (29.4)	
Anxiety and/or depression			< 0.001			0.003
No	176 (81.1)	176 (96.7)	(0,241)	266 (85.0)	98 (96.1)	(0.145)
Yes	41 (18.9)	6 (3.3)		47 (15.0)	4 (3.9)	
AUDIT			0.434			0.607
0–5	161 (85.2)	144 (88.3)	(0.046)	236 (85.8)	81 (88.0)	0.028)
6-	28 (14.8)	19 (11.7)		39 (14.2)	11 (12.0)	
Work-family conflict, 0–5 years			< 0.001			0.001
No	152 (72.4)	162 (89.5)	(0,215)	235 (77.0)	92 (92.0)	(0.163)
Yes	58 (27.6)	19 (10.5)		70 (23.0)	8 (8.0)	
Work-family conflict, 6–12 years			< 0.001			0.008
No	141 (67.5)	161 (89.0)	(0.256)	228 (74.8)	87 (87.9)	(0.136)
Yes	68 (32.5)	20 (11.0)		77 (25.2)	12 (12.1)	
Work-family conflict, 13–20 years			< 0.001			0.001
No	136 (65.4)	154 (86.0)	(0.238)	216 (71.5)	87 (87.9)	(0.164)
Yes	72 (34.6)	25 (14.0)		86 (28.5)	12 (12.1)	
Stressful life event			< 0.001			0.136
No	72 (33.3)	95 (52.5)	(0.193)	126 (40.5)	50 (49.0)	(0.074)
Yes	144 (66.7)	86 (47.5)		185 (59.5)	52 (51.0)	

Health status: *Good health* = self-reported health said to be excellent or very good while also having a total score ≤ 4 on the PHQ-scale and *Poor health* = health to be less good or poor, or having a totals score ≥ 5 on the PHQ-scale

Major life events include: Illness/accident – husband/wife/partner/child, Death - husband/wife/partner/child, Death - close relative/friend, Worsened household finances

*16 cases of missing value on Health status

**Fig. 2:**
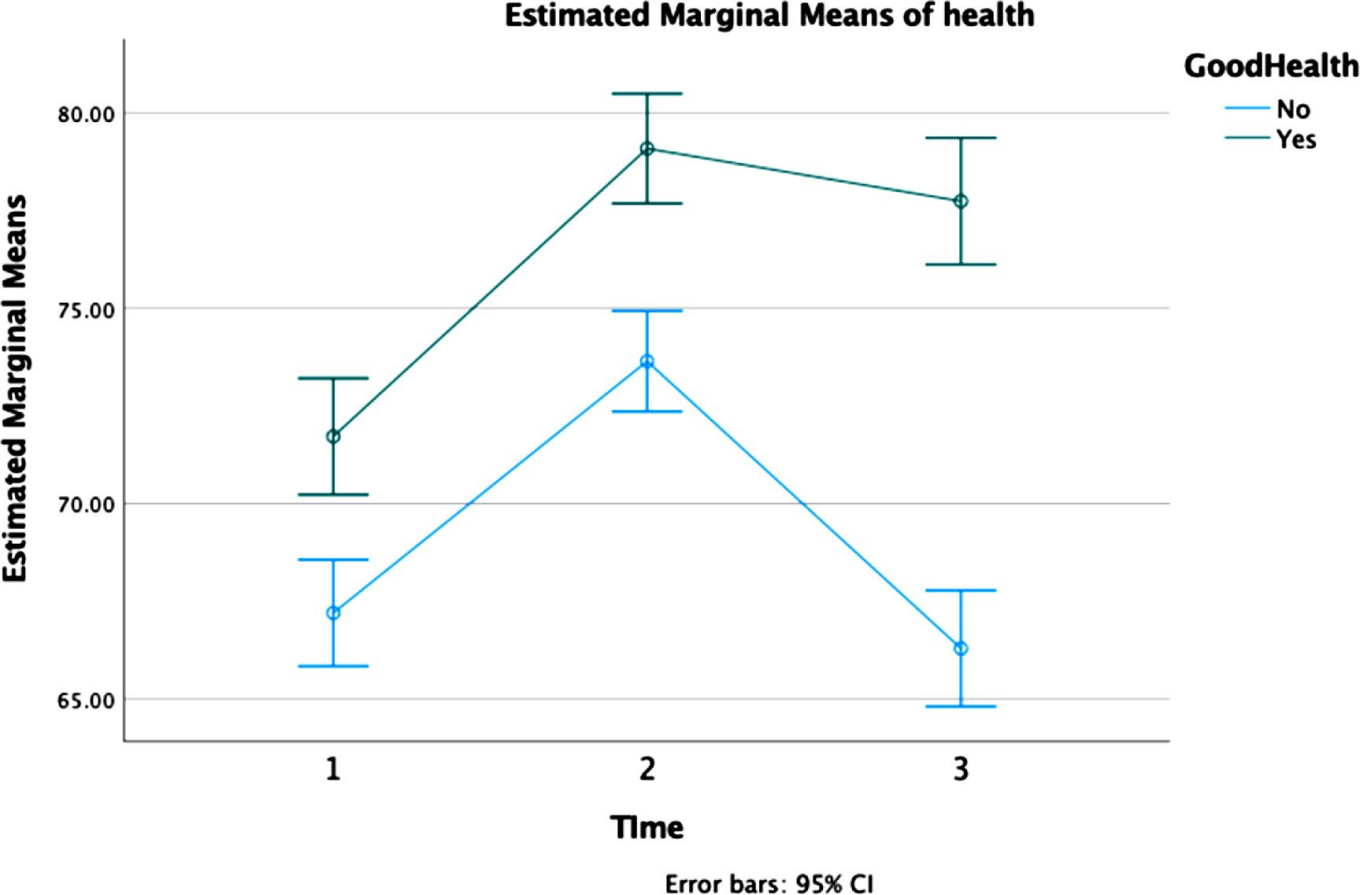
Sense of coherence at three timepoints by health status 25 years after inclusion. *Note*: 1 = 3 years after inclusion; 2 = 12 years after inclusion, 3 = 25 years after inclusion

**Fig. 3:**
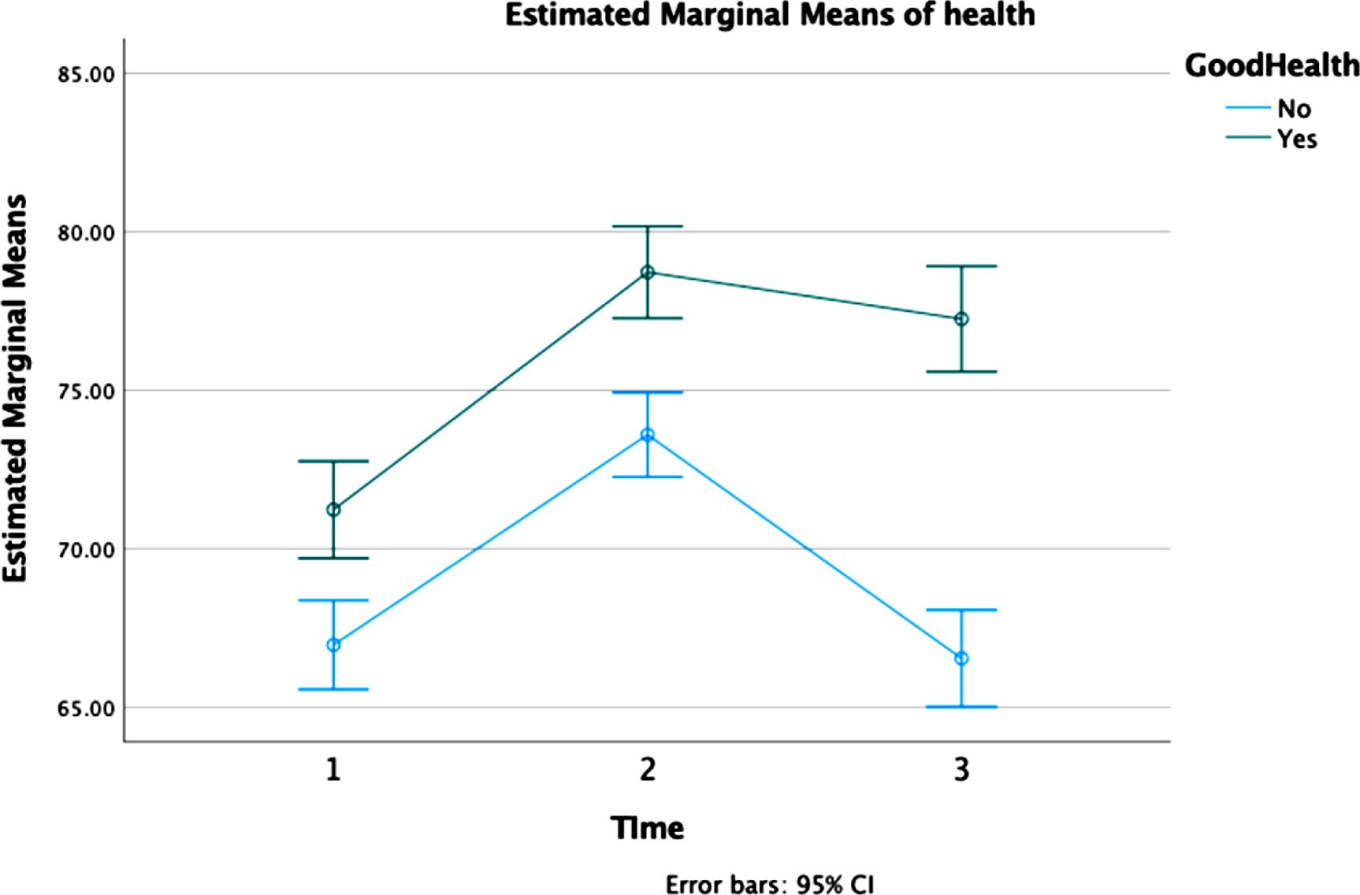
Sense of coherence at three timepoints by health status 25 years after inclusion, adjusted for divorce and life events. *Note*: 1 = 3 years after inclusion; 2 = 12 years after inclusion, 3 = 25 years after inclusion

Descriptive data on SOC at the three different timepoints indicate an increase of SOC total score between three and 12 years after inclusion. However, between the years 12 and 25 SOC decreases some, though remains at a higher level compared at three years. Further analyses, adjusting for health status, confirmed this pattern and also showed that women of good health have a higher SOC-score at all three measurements compared to women of poor health. The analyses also showed that overall, 7.5% of the women scored above the 75th percentile at all three measurements. However, scoring above the 75th percentile was more common among women with a good SRH compared to women reporting poor health (2.3% vs. 14.3%, p < 0.001, effect size = 0.308), Table 2. A sharp increase between 1st and 2nd measurement was present among women of both good and poor health. However, the score among women of good health remains stable just under 80 at measurements at both 12- and 25-year follow-ups, whereas women of poor health drops sharply bringing their score below the level at the first follow-up, Fig. 2. When adjusting scores by EPDS, having been through a divorce or experienced life events, the difference between the group was marginally reduced, Fig. 3.

**Table 2:** Crosstabulation of edinburgh postnatal depressions scale (EPDS), sense of coherence (SOC) at 3-, 12-, and 25-years, and health status 25 years after inclusion

	Health status			SOC 25 years		
	Poor	Good		<=75th percentile	> 75th percentile	
	n (%)	n (%)	p-value* (Effect size)	n (%)	n (%)	p-value* (Effect size)
EPDS (cut 10)			0.013			0.010
< 10	194 (89.4)	174 (96.1)	(0.127)	281 (90.1)	100 (98.0)	((0.127)
> 10	23 (10.6)	7 (3.9)		31 (9.9)	2 (2.0)	
SOC stability			< 0.001			< 0.001
Never > 75th percentile	151 (69.6)	77 (42.3)	(0.308)	227 (72.5)	8 (7.8)	(0.622)
Mixed above or below	61 (28.1)	79 (43.4)		82 (26.2)	64 (62.7)	
Always > 75th percentile	5 (2.3)	26 (14.3)		4 (1.3)	30 (29.4)	
SOC 3 years			< 0.001			< 0.001
<=75th percentile	185 (85.3)	129 (70.9)	(0.175)	271 (86.6)	54 (52.9)	(0.351)
> 75th percentile	32 (14.7)	53 (29.1)		42 (13.4)	48 (47.1)	
SOC 12 years			< 0.001			< 0.001
<=75th percentile	186 (85.7)	119 (65.4)	(0.239)	266 (85.0)	49 (48.0)	(0.372)
> 75th percentile	31 (14.3)	63 (34.6)		47 (15.0)	53 (52.0)	
SOC 25 years			< 0.001			
<=75th percentile	194 (89.4)	107 (58.8)	(0.345)			
> 75th percentile	23 (10.6)	75 (41.2)				

Health status: *Good health* = self-reported health said to be excellent or very good while also having a total score ≤ 4 on the PHQ-scale and *Poor health* = health to be less good or poor, or having a totals score ≥ 5 on the PHQ-scale

*Fisher’s exact test

Analyses of the symptom stability of SOC in relation to health status 25 years after inclusion showed that having scored above the 75th percentile on SOC on all three occasions were more common among women who reported good health compared with women who reported poor health, Table 2. On the other hand, never having scored above the 75th percentile was almost twice as common among women with poor health compared to women with a good health, Table 2. Moreover, women who scored above the 75th percentile on SOC 25 years after inclusion were more likely to have scored above the 75th percentile on SOC in previous follow-ups, Table 2. Also, not exhibiting symptoms of PPD three months after childbirth was associated with a good health as well as a high level of SOC.

Further analyses on symptom stability suggested that the women who scored high on SOC at all measurements were more often born in Sweden (8.4% vs. 4.2%, (Not statistically significant (NS)) and had never been through a divorce (10.6% vs. 4.0%, p = 0.040). Similarly, women with a history of anxiety were appeared to be more prone to have never scored above cut-off on SOC (72% vs. 56%, NS) as were women with a history of depression (74% vs. 55%, p = 0.026) and fatigue syndrome (82% vs. 56%, NS). Regarding women who have scored high on one or two occasions no clear pattern was detected. However, women scoring below the 75th percentile at all three occasions reported poorer health.

### Regression analyses

The single logistic regression models, presented in Table 3, showed that women had an increased likelihood of reporting good health if they had a college/university degree (OR = 1.85, 95% CI = 1.24–2.75), had not been through a divorce (OR = 1.78, 95% CI = 1.18–2.68), did not report anxiety/depression at the 25 year follow-up (OR = 6.83, 95% CI = 2.83–16.50), and had not experienced stressful life events (OR = 2.21, 95% CI = 1.47–3.32). In general, the effect sizes were small. However, work family conflicts and symptoms of anxiety and/or depression provided medium to large effect sizes. Similar results were found when analyzing SOC at the 25-year follow-up. Women who had not been through a divorce (OR = 1.92 95% CI = 1.18–3.10), and women who did not report anxiety/depression at the 25-year follow-up (OR = 4.33, 95% CI = 1.52–12.33) had an increased likelihood of having a SOC score above the 75th percentile at the 25-year follow-up, while stressful life events were not associated with SOC, Table 3. In addition, not having experienced work-family conflict during their children’s upbringing increased the likelihood of both reporting good health and scoring above the 75th percentile on SOC at the 25-year follow-up. As regarding Good health, most associations did not have large effect sizes though reaching statistical significance.

**Table 3:** Crude odds ratio (cOR) and corresponding 95% confidence intervals (CI) for good health and SOC

	Good health		SOC 25 years > 75th percentile	
	cOR (95%CI) n = 352–399	Effect size	cOR (95%CI) n = 415	Effect size
**Region of birth**				
Sweden	1.20 (0.51–2.85)	0.077	0.80 (0.29–2.19)	-0.007
Outside Sweden	Reference		Reference	
**Marital status**				
Single	Reference		Reference	
Married/cohabiting	1.60 (0.77–3.35)	0.199	1.17 (0.51–2.68)	0.014
Divorced/Widow	1.53 (0.57–4.08)	0.180	0.56 (0.18–1.93)	-0.045
**College/university**				
No	Reference		Reference	
Yes	1.85 (1.24–2.75)	0.261	1.09 (0.70–1.70)	0.010
**Any divorce**				
No	1.78 (1.18–2.68)	0.245	1.92 (1.18–3.10)	0.069
Yes	Reference		Reference	
**Anxiety and/or depression**				
No	6.83 (2.83–16.50)	0.815	4.33 (1.52–12.33)	0.519
Yes	Reference		Reference	
**AUDIT**				
0–5	1.32 (0.71–2.46)	0.118	1.22 (0.60–2.49)	0.010
6-	Reference		Reference	
**Work-family conflict, 0–5 years**				
No	3.25 (1.85–5.72)	0.500	3.43 (1.59–7.40)	0.268
Yes	Reference		Reference	
**Work-family conflict, 6–12 years**				
No	3.88 (2.25–6.71)	0.575	2.45 (1.27–4.72)	0.224
Yes	Reference		Reference	
**Work-family conflict, 13–20 years**				
No	3.26 (1.96–5.43)	0.502	2.89 (1.50–5.55)	0.234
Yes	Reference		Reference	
**Stressful life event**				
No	2.21 (1.47–3.32)	0.336	1.41 (0.90–2.21)	0.050
Yes	Reference		Reference	

Single logistic regression, where health status and SOC at 25 years are modelled separately

Health status: *Good health* = self-reported health said to be excellent or very good while also having a total score ≤ 4 on the PHQ-scale and *Poor health* = health to be less good or poor, or having a totals score ≥ 5 on the PHQ-scale

Multiple logistic regression on good health showed that the likelihood of reporting good health increased with the number of times the women had reported SOC-scores above the 75th percentile (1–2 times OR = 2.57 95% CI = 1.64–4.03, effect sizw = 0.401, 3 times OR = 9.82 95% CI = 3.55–27.18, effect size = 0.965) compared to never having scored above the 75th percentile, Table 4. Women who had not been through a divorce were close to 60% more likely to report good health compared to women who had been through a divorce, whereas women not having experienced stressful life events during the past two years were more than twice as likely to report good health (OR = 2.35, 95% CI = 1.52–3.63, effect size = 0.363) compared to women who had experienced stressful life events, Table 4. Regarding SOC it appears as if symptoms of PPD have some lingering effect at the 25-year follow-up, Table 4. Including concurrent symptoms of anxiety and/or depression in the regression model did not alter this finding (data not shown). In addition, not having been through a divorce increased the likelihood of SOC scores above the 75th percentile (OR = 1.82, 95% CI = 1.12–2.96, effect size = 0.050) whereas stressful life events did not have any effect on SOC, Table 4.

**Table 4:** Adjusted odds ratio (aOR) and corresponding 95% confidence intervals (CI) for good health, and SOC

	Good health		SOC 25 years > 75th percentile	
	aOR (95%CI)*	Effect size	aOR (95%CI)***	Effect size
**SOC stability**				
Never > 75th percentile	Reference		NA	
Mixed above or below	2.57 (1.64–4.03)	0.401		
Always > 75th percentile	9.82 (3.55–27.18)	0.969		
**EPDS (cut 10)**				
< 10	2.19 (0.88–5.48)	0.333	5.22 (1.22–22.34)	0.239
> 10	Reference		Reference	
**Any divorce**				
No	1.57 (1.01–2.44)	0.191	1.82 (1.12–2.96)	0.050
Yes	Reference		Reference	
**Stressful life events**				
No	2.35 (1.52–3.63)	0.363	1.43 (0.90–2.25)	0.056
Yes	Reference		Reference	

*Multiple logistic regression, adjusted for all factors presented in Table 4

**EPDS excluded from analysis due to singularities in the model (all of the women with > 10 were below 75th)

***Adjusted for EPDS, any divorce, and life events

## Discussion

This study investigated the associations between SOC and self-rated health in 415 women followed from giving birth until 25 years post childbirth. The first research question considered whether SOC and self-rated health (SRH) were related during the 25 years of follow-up. Among women of both good and poor health, SOC was found to increase between the 3-year and the 12-year follow-up, followed by a decrease in SOC between the 12-year and the 25-year follow-up. Dziuba et al. (2021) showed increasing scores over 10 and 20 years respectively [15], while decreasing SOC scores have been found in a Swedish population-based study with a five-year follow-up period [8]. Among women reporting good health the decrease of SOC was quite small and the SOC remained at a higher level at the 25-year follow-up than at the 3-year follow-up. Among women reporting poor health, the decrease was substantial and bringing the SOC to a level below the SOC at the 3-year follow-up. This finding could be due to several causes. Firstly, it is possible that women of good health are better equipped to handle unexpected and stressful events compared to women of poor health as studies have shown that women with a high SOC are more likely to maintain a high level of SOC in subsequent follow-ups compared to women who score lower on the first measurement of SOC. Secondly, previous studies have also shown that women with a high SOC are more likely to make healthier choices thus further promoting a better health [4]. In the present study, a clear association between stable high SOC scores and good SRH was found. This finding is in line with a systematic review on SOC and health, showing a strong association between SOC and perceived good health, indicating that SOC has main as well as mediating and moderating effects on health [3].

The second research question investigated associations between postpartum depression and SOC and SRH respectively, later in life. Univariately PPD had an impact on SOC at all measurements, adjusting for other factors the results were no longer statistically significant. Some lingering effect of EPDS was found on SOC at the 25-year follow-up, though no association remained with SRH. Moreover, when concurrent symptoms of depression and anxiety was controlled for the lingering effect of EPDS remained. It is plausible that PPD predisposes for subsequent depression as previous studies have indicated. A Swedish population based study on 333 mothers showed a significant association between PPD and SOC three months postpartum [31], and other studies have indicated persistent depressive symptoms at three and five years postpartum when having experienced preterm birth [32, 33]. Also, it has been shown that symptoms of depression, measured using EPDS, may remain as long as 16–17 years post childbirth [34].

The third research question focused on how life events, depression/anxiety, educational level, family situation, and work/family conflicts are associated with SOC and SRH 25 years post childbirth. The current study found that higher level of education and not reporting stressful life events during the past two years were associated with reporting a better health whereas no association was found with respect to SOC. This is both in line and in contrast to previous findings reported by other studies, where a higher level of education has been shown to have either no impact or a positive impact on SOC [35, 36].

In the present study, experience of divorce was associated with both SRH and SOC. This finding is in line with previous results indicating higher levels of illness a decade after divorce, as well as higher levels of stressful events [37]. Women in the present study are all mothers and thus possibly affected by a divorce both in terms of increased workload and worsened household finances.

As several studies have indicated, women who have experienced excessive workload or family/work conflicts have been found to have a lower SOC as well as a worse health. Similar findings were found in the current study, where women who had not had any of these experiences reported a somewhat higher SOC but reported a considerable better health status. This may be explained by the reduced level of stress these women have experienced, as a high level of stress has been found to have negative effects on health. It could also be due to the high SOC itself, as a high SOC promotes better health choices and may have helped the women to make healthy choices regarding work/family allocation during the child’s upbringing.

As a high SOC has been found to promote better health, but also that experiencing a good health is related to a higher SOC it is hard to decipher the actual causal direction. It is plausible that high SOC and a good SRH are interconnected and thus explains why women with a high SOC are more likely to maintain their high SOC.

### Strengths

A major strength of the current study is its long follow-up time, approximately 25 years. Also, given the long follow-up the study population remains relatively large. Also, the use of standardized, and validated instruments further strengthens the study.

### Limitations

Limitations of the current study includes a high non-response compared to baseline which may affect the generalizability of the findings. Especially since “vulnerable” women are less likely to have participated in the current follow-up. In the current study the 75th percentile was used as a cut-off on SOC, as scores above the 75th percentile captures women who experience a high sense of coherence. In addition, in comparison to previous studies [38, 39] the women generally scored high on SOC - overall mean = 70.5, and among women with less god SRH mean = 65.0 whereas women with good SRH had a mean score of 77.1. However, this under-representation of vulnerable women can be expected to lead to an underestimation of found differences. These limitations combined with rather wide confidence intervals of the estimates are important factors when interpreting the results. Also, statistical significance does not imply a large effect size, i.e., the standardized difference between groups may still be rather small or not clinically significant. In the current study the effect sizes were generally small, except for symptoms of depression and/or anxiety, experience of stressful life events and work-family conflicts. Other limitations of the study is the effect the limited study population has on the possibility to apply more advanced statistical methods and number of factors that can be included in the multivariable analyses and the use of psychometric scales in a Swedish setting.

## Conclusion

The findings in the current study are in line with other studies exploring a predictive value of SOC and self-perceived health. A stronger and more stable SOC can be found among women with better health. We have studied women over a 25-year period and the vast majority of the women who took part in this study were stable in their assessments indicating that these women are, generally, of good health and with a good SOC. Moreover, the study findings also indicate that an early identification of depressive symptoms after childbirth as well as a low sense of coherence may be beneficial for women who may need extra support to increase their long-term health and sense of coherence.

## Data Availability

The data analyzed and supporting the findings of the current study are not publicly available. Data are however available from the corresponding author upon reasonable request.

## List of abbreviations

AUDIT: Alcohol Use Disorders Identification Test
CI: Confidence interval
EPDS: Edinburgh postnatal depression scale
GAD: Generalized Anxiety Disorder
OR: Odds ration
PHQ: The Patient Health Questionnaire
PPD: Postpartum depression
SOC: Sense of coherence
SRH: Self-reported health
